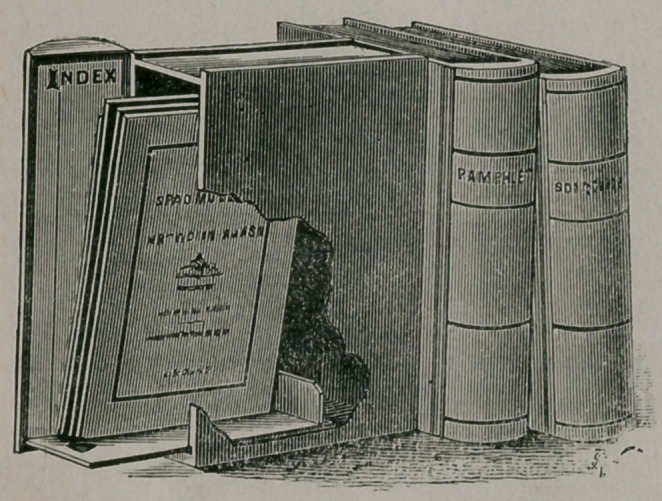# Zoological Garden—Infected Cattle—Personal—New State Board of Health—Hog Cholera—Extent of Lung Disease—Inoculation of Rabies—Domestication of Certain Ruminants, &c.—A New Theory Concerning Gout

**Published:** 1880-07

**Authors:** 


### MISCELLANEOUS.

---

ZOOLOGICAL GARDEN, CENTRAL PARK —Bison cow gave birth on May 14th to a 100 lb. bull calf, which is at present alive and doing well. Two zebu cows also have given birth to calves, both bulls, one March 24th, the other on May 7th, this year. The camel, now seven years old, gave birth to a fine female calf on April 4th—term of gestation just twelve months.

---

INFECTED CATTLE.—The Conference Committee of the Senate and Assembly to which was referred the Supply bill at the close of the session having left out the appropriation for carrying on the work of the State Cattle Commission no aggressive work can be carried on this year, and General Patrick, the Commissioner, says that he is instructed by the Governor to hold all infected districts in quarantine, to prevent New York and the non-infected counties from receiving cattle from infected districts, and by all possible means to prevent the spread of the disease. To accomplish this object the police authorities of the city of New York have issued orders to prevent cattle from being landed in or removed from the city without its permit.

Fresh cows will continue to arrive by rail and the steamers from the river counties as heretofore, and all restrictions upon the movements of cattle within the city limi s are withdrawn.

On Long Island the district east of a north and south line through Jamaica is now free from disease.

That portion of the island lying west of Jamaica, including Brooklyn, Long Island City, Newtown, etc., being considered as an infected district, no cattle will be allowed to be removed across said line to t e eastern district without permit, and t e police of the ity of Brooklyn and Long Island City have issued orders to prevent any milk cows or store cattle from coming to or leaving the district by water. All restrictions upon the movement of cattle within the city of Brooklyn, Long Island City and all the district west of Jamaica are withdrawn.

Tne St tes of New Jersey, Pennsylvania and Maryland, the northern end of Staten Island and the western end of Long Island, being regarded as infected districts, the reason fo these restrictions is obvious.

To obtain permits in extraordinary cases for crossing these lines, or making any exception to these restrictions, applications must be made at the office of the Commission, Argus Building, No. 29 Fulton street, Brooklyn.

The Sheriffs of the counties interested (outside of the cities) are instructed to prevent and punish the violation of chapter 134, Laws of 1878, in relation to contagious diseases among cattle.

GROWTH AS A FUNCTION OF CELLS —Dr. Charles Sedgwick Minot has published an article in the Proceedings of the Boston Society of Natural History on "Growth as a Function of Cells." This essay is an attempt to give an exact analysis of the problem of growth. The author considers that growth depends upon an impulse created at the time when the *ovum* is impregnated; this impulse he terms rejuvenation, because the vital power is made young again in a new cycle of cells. The old cycle of cells passes away, the parent dies, but a new egg-cell is produced endowed with an extraordinary power of division, which causes the birth of successive generations of cells. Now usually the number of cells is doubled at every division, that being the least possible increase, hence the number of cells must increase in geometrical progression; therefore, the growth of every animal would be indefinite were there not an opposing influence. This opposing influence cannot be the loss of a part of the cells, as when part of the skin peels off, for this loss is too slight to counterbalance the multiplication. The explanation is, that the intervals between the births of two successive generations of cells continually increases, or in other words the frequency of the divisions continually diminishes. This Dr. Minot calls the phenomeon of *senescence*, to which he attributes the utmost importance, as a vital phenomen common to all animals, yet hitherto entirely unstudied. He says: "From our point of view this change (in the frequency of division) is the most important alteration produced by senescence; that it really occurs is not only a deduction, but is shown by actual observation, for no one can question that the division of the cells during segmentation of the yoke proceeds at shorter intervals than during adult life; thus in an egg say eight or ten, perhaps more, generations of cells may be born in the course of a single day, all the cells dividing; but we cannot for an instant imagine that all the cells of the human adult, for example, divide upon an average even once a day, probably . . . . . not . . . . even once a year." But the size or weight of the whole animal depends not only upon the number but also on the volume and weight of the cells. Dr. Minot therefore discusses the laws which govern the variations of the size of cells. The relations of growth to the size of animals is next considered, the conclusion being drawn that the rapidity of the senescence determines the size of the animal, because the more rapidly the frequency of the cell divisions diminishes, the sooner will growth cease and the smaller will the animal remain, so that in this respect senescence exercises a fundamental influence. This is, we believe, the only scientific attempt to explain the reason why animals are of different sizes. Finally, by a novel reasoning, the conclusion is drawn, that although the animal grows in three dimensions, yet the growth of the cells is confined to two dimensions of space. For the detailed arguments supporting the author's conclusions, the original article must be consulted.

---

THE NEW STATE BOARD OF HEALTH.—The bill creating a State Board of Health has become a law, and the members are already appointed. These are Dr. Elisha Harris, John S. Delavan, and Erastus Brooks. As representatives of City Boards already existing, the Governor has appointed Dr. Charles F. Chandler, of New York, Dr. James G. Hunt, of Utica, and Dr. Edward M. Moore, of Rochester. The other three members are the Attorney-General, the Superintendent of the State Survey, and the Health Officer of the Port of New York.

Cows will quickly fall off in their milk unless they have plenty of water within convenient reach. They will suffer considerably from thirst before they will travel long distances for water, either in hot or cold weather. If left too long without water, until they become feverish. they will drink too much, and this going from one extreme to the other will affect unfavorably the health of the cow, and cause a decrease of milk. Cows are kept solely for producing milk, and any unnecessary exertion that they are obliged to make to procure food or drink will divert so much of their energy from milk production.—*Nebraska Farmer.*

---

EXTENT OF THE LUNG PLAGUE.—The report of Dr. C. P. Lyman, recently made to the Commissioner of Agriculture, shows that contagious pleuro-pneumonia now exists in one county of Connecticut, five counties of New York fourteen counties of New Jersey, ten counties of Pennsylvania, four counties in Maryland. There are cases, also, in the District of Columbia and in Virginia.

---

Hog cholera in a strange and most malignant form has broken out among the swine of farmers in the vicinity of Oconomowoc, Wis. One large stock-raiser in Summit lost nearly one hundred fine hogs in less than two weeks by means of the epidemic —*Nebraska Farmer.*

---

E. C Wendt, M D., has been appointed to the Chair of Comparative Anatomy, Embryology. etc., Columbia Veterinary College.

---

A NEW PAMPHLET BOX.—T. L. Clacher, of New York, 107 East Twenty-Eighth St., has devised a novel pamphlet box. It is a very simple and convenient receptacle for pamphlets, journals, manuscripts, etc. It is made to represent a handsomely bound book, although in reality it is a box. The back closes with a spiring, and works by a hinge arrangement. It shuts of its own accord. The bottom on which the pamphlets rest is movable, and when drawn out brings the contents of the box with it, and also serves to keep the lid open, so that it is possible to consult the contents of the box without removing it from the shelf. It is made in different sizes to accommodate any kind of a periodical. On the inside of the lid is the index.

## Figures and Tables

**Figure f1:**